# Blood Clots May Compromise Intracranial Pressure Measurement Using Air-Pouch Intracranial Pressure Probes

**DOI:** 10.3390/jcm12113661

**Published:** 2023-05-25

**Authors:** Sae-Yeon Won, Sascha Herrmann, Daniel Dubinski, Bedjan Behmanesh, Svorad Trnovec, Nazife Dinc, Joshua D. Bernstock, Thomas M. Freiman, Florian A. Gessler

**Affiliations:** 1 Department of Neurosurgery, University Rostock, 18057 Rostock, Germanydaniel.dubinski@med.uni-rostock.de (D.D.); florian.gessler@med.uni-rostock.de (F.A.G.); 2 Department of Neurosurgery, University Hospital Jena, 07743 Jena, Germany; 3 Department of Neurosurgery, Brigham and Women’s Hospital, Harvard Medical School, Boston, MA 02115, USA; 4 Department of Mechanical Engineering, Massachusetts Institute of Technology, Cambridge, MA 02139, USA

**Keywords:** intracranial pressure (ICP) monitoring, probe placement, air-pouch balloon-assisted probe

## Abstract

**Background**: Air-pouch balloon-assisted probes have proven to be both simple and reliable tools for intracranial pressure (ICP) monitoring. However, we experienced reproducible falsely high ICP measurements when the ICP probe was inserted into the intracerebral hematoma cavity. Thus, the aim of the experimental and translational study was to analyze the influence of ICP probe placement with regard to measured ICP values. **Methods**: Two Spiegelberg 3PN sensors were simultaneously inserted into a closed drain system and were connected to two separate ICP monitors thereby allowing for simultaneous ICP measurements. This closed system was also engineered to allow for pressure to be gradually increased in a controlled fashion. Once the pressure was verified using two identical ICP probes, one of the probes was coated with blood in an effort to replicate placement within an intraparenchymal hematoma. Pressures recorded using the coated probe and control probe were then recorded and compared across a range of 0–60 mmHg. In an effort to further the translational relevance of our results, two ICP probes were inserted in a patient that presented with a large basal ganglia hemorrhage that met criteria for ICP monitoring. One probe was inserted into the hematoma and the other into brain parenchyma; ICP values were recorded from both probes and the results compared. **Results**: The experimental set-up demonstrated a reliable correlation between both control ICP probes. Interestingly, the ICP probe covered with clot displayed a significantly higher average ICP value when compared to the control probe between 0 mmHg and 50 mmHg (*p* < 0.001); at 60 mmHg, there was no significant difference noted. Critically, this trend in discordance was even more pronounced in the clinical setting with the ICP probe placed within the hematoma cavity having reported significantly higher ICP values as compared to the probe within brain parenchyma. **Conclusions**: Our experimental study and clinical pilot highlight a potential pitfall in ICP measurement that may result secondary to probe placement within hematoma. Such aberrant results may lead to inappropriate interventions in an effort to address falsely elevated ICPs.

## 1. Introduction

Intracranial pressure (ICP) monitoring is critical in the clinical management of patients with intracranial insults/lesions [1,2,3]. Monitoring with intracerebral pressure probes has become more prevalent as compared to ventricular drain measurements (i.e., the “gold standard”) [4,5,6]. It is however prudent to note that no consensus exists with regard to which ICP monitoring modality is ultimately employed clinically [1]. There are several types of intracerebral pressure sensors (e.g., fiberoptic or stain-gauge); these sensors require calibration procedures and carry the risk of a silent zero drift [7]. To overcome such limitations, an air-pouch balloon-based sensor was introduced ~20 years ago by Spiegelberg (Aesculap, Inc., Center Valley, PA, USA) [8]. One of the benefits of this probe is the automatic zeroing after connection to the monitoring unit; this process is repeated every hour reducing the risk of a silent zero drift [8]. Previous work has proven that Spiegelberg air-pouch measurements are reliable and correlate with ICPs ascertained via a ventricular drain [8,9].

Despite the promise of Spiegelberg air-pouch balloon-based ICP measurements, herein we present a clinical scenario in which we were misled by discordant ICP values secondary to clot adherence on the ICP probe. Based on this experience, we sought to clarify our initial clinical findings experimentally (i.e., at the bench) and ultimately returned to the bedside in an effort to validate our experimental findings (i.e., via a clinical pilot).

### Case Illustration

**Figure 1:**
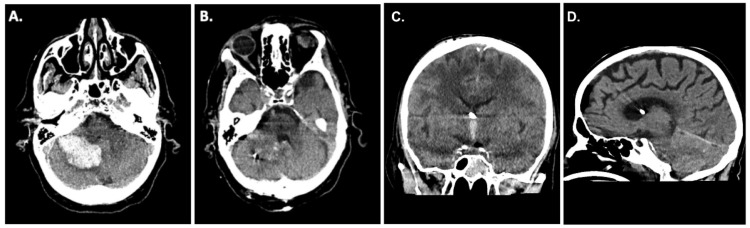
(**A**) Preoperative CT scan with the diagnosis of a spontaneous cerebellar hemorrhage on the right side. (**B**) CT scan after hematoma evacuation and insertion of a Spiegelberg 3-PN sensor in the cerebellar parenchyma on the right side. (**C**) Postoperative CT scan showing the correct location of the combined external ventricular drain-ICP probe in the right ventricle. (**D**) CT scan after hematoma evacuation showing no sign of transtentorial herniation.

A 62-year-old patient presented to the emergency room at our institution after collapsing. Due to a worsening mental status, he was electively intubated and a computed tomography (CT) scan of the brain revealed a cerebellar hemorrhage with compression of the fourth ventricle/resultant hydrocephalus (Figure 1A). CT angiography failed to demonstrate any underlying vascular pathology. Due to the size of the hemorrhage and its mass effect, the patient was taken for an emergency clot evacuation and surgical decompression. Just prior to the decompression, a combined external ventricular drain-ICP probe from Spiegelberg was placed in the right frontal ventricle; the sub-occipital craniotomy with hematoma evacuation was uncomplicated. During the case, an additional infratentorial ICP probe was inserted into the hematoma cavity as per our institutional standard. After replacing the bone, the infratentorial probe exhibited an ICP value of 27 mmHg, whilst the supratentorial probe reported 13 mmHg. Despite several attempts to re-zero and connect to another ICP monitor, the infratentorial ICP value did not change. As such, we felt it was prudent to explore the surgical site, yet upon reopening, the surgical field showed no sign of rebleeding and/or massive swelling of the cerebellum. Even the cerebellum was under the level of the dura, which was discordant with the elevated ICP value of 27 mmHg. We removed the ICP probe, and a new ICP probe was inserted into the cerebellum (Figure 1B). Observing the previous ICP probe, we noticed a thin clot adhering to the air-pouch of the ICP probe. After revising the probe’s position, the infratentorial ICP was noted to be 15 mmHg. The postoperative CT scan showed sufficient hematoma evacuation with proper location of the combined EVD-ICP probe in the right ventricle (Figure 1B,C). In addition, there was no sign of transtentorial herniation in the postoperative CT scan (Figure 1D). The remainder of the patient’s post-operative course was uneventful with reliable ICP recordings, and they were ultimately discharged to a rehabilitation facility with residual imbalance and dysmetria.

## 2. Methods

The study was approved by the ethics committee, [BLINDED FOR REVIEW]. Formal consent of the patients described/presented within the text were obtained. The majority of the reported work involved an experimental study/apparatus (i.e., no animal or human involvement).

### 2.1. Experiment Setting

**Figure 2:**
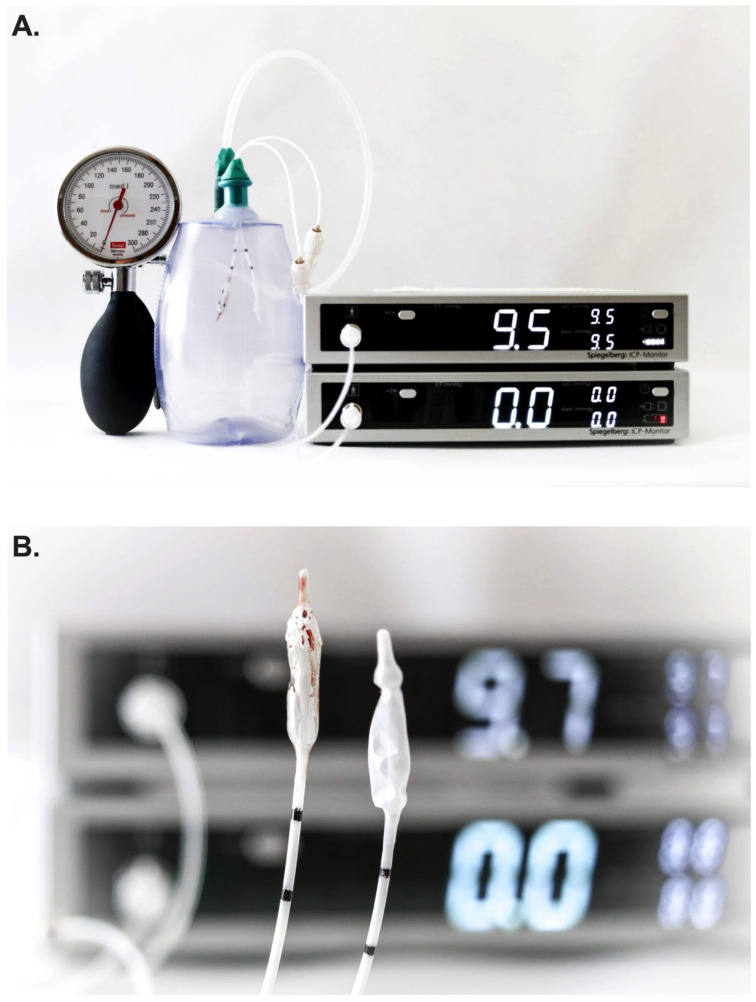

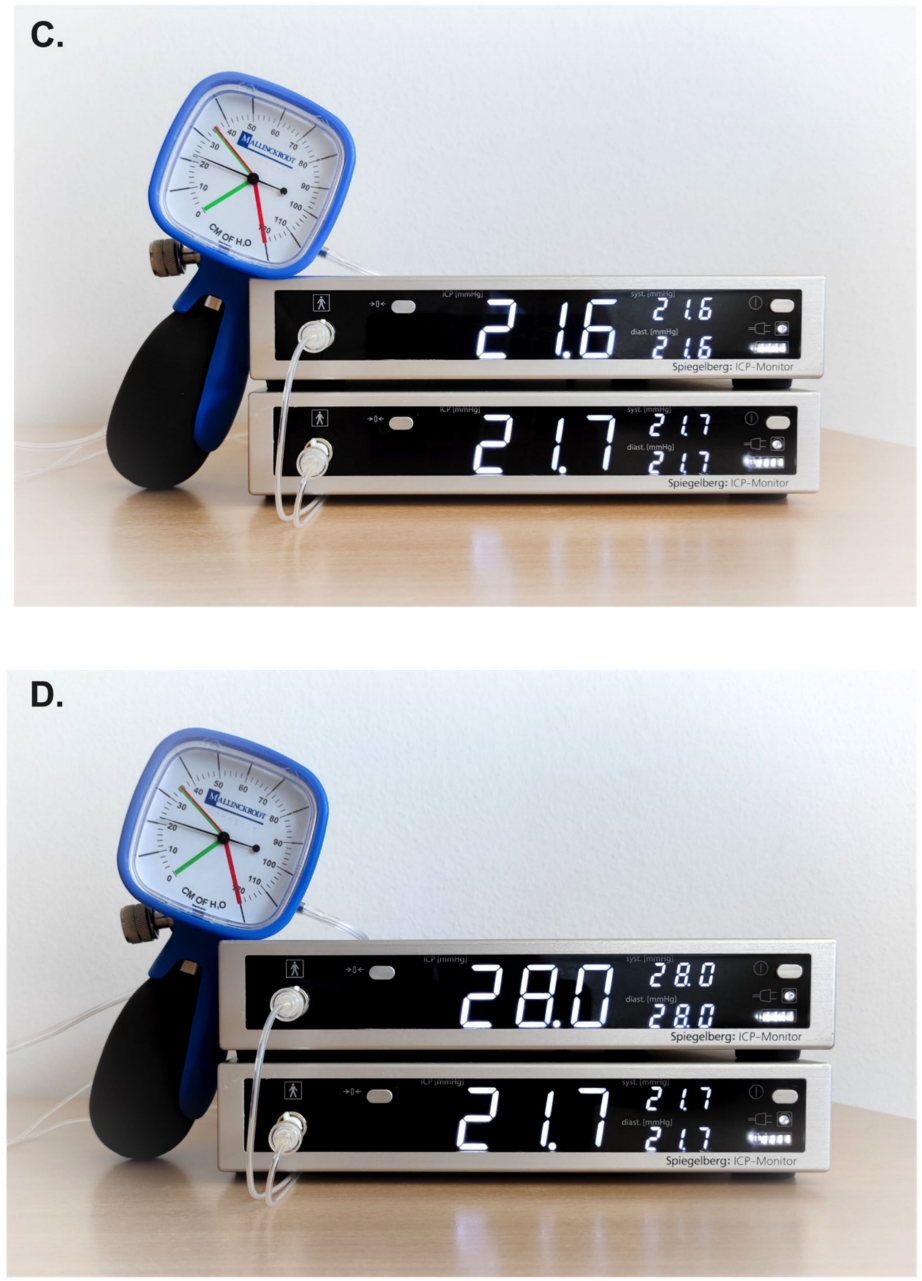
(**A**) Experimental setting with simultaneous measurement of two Spiegelberg 3-PN sensors. (**B**) The left Spiegelberg 3-PN sensor shows a clot adhesive to the air-pouched balloon. The right sensor serves as a control. (**C**) In the control group, both ICP monitors show similar ICP values which is in accordance with the pressure measured by the manometer (approximately 21 mmHg). (**D**) In the experimental group, the control ICP probe still shows 21.7 mmHg in accordance with the pressure measured by the manometer; however, the clot-coated ICP probe shows a higher error value of 28 mmHg. ICP, intracranial pressure.

Two Spiegelberg 3PN sensors were simultaneously inserted into a closed drain system and were connected to two separate ICP monitors, thereby allowing for simultaneous ICP measurements (PRIVAC@, Primed Halberstadt, Medizintechnik GmbH, Halberstadt, Germany). Through a separate port, a 12F tube was connected to the drain system and a blood pressure manometer (BOSO, BOSCH + SOHN GmbH u. Co.KG, Jungingen, Germany). In so doing, the pressure within the closed drain system could be altered in a controlled manner (Figure 2A).

### 2.2. Study Design

Two Spiegelberg 3PN sensors were inserted into the closed drain system, and a simultaneous measurement of pressure was performed during steady increases in pressure within the chamber (i.e., from 0 to 30 mmHg in 5 mmHg increments and from 30 to 60 mmHg in 10 mmHg increments). To minimize potential sources of bias, this experiment was repeated >5 times on different days by two independent investigators (S.W. and S.H.).

Once the system was validated and the concordance of measurements between probes confirmed in the experiment setting, one Spiegelberg 3PN sensor was placed into a Petri dish filled with 5 mL of fresh whole human blood obtained from the hospital’s laboratory core just prior to the experiment. After waiting for coagulation (~30 min), a thin layer of clot was subsequently visible on the air-pouch balloon sensor (Figure 2B). Thereafter, simultaneous measurements of pressure were again performed as per the above using both ICP probes in the experimental setting (i.e., clot-layered and control).

### 2.3. Translation of Experimental Data (Pilot Clinical Case)

Given our index case and the results obtained during our experimental study, we felt it was critical to attempt to validate our findings in a relevant clinical setting.

A 50-year-old patient was admitted to our hospital secondary to hypertensive intracerebral hemorrhage within the basal ganglia. After consent, the patient was taken to the operating room for the surgical placement of two ICP monitoring devices. One air-pouch probe was placed within the hematoma cavity, and the other one in the adjacent cerebral parenchyma. After surgery, the patient was transferred to the neuro-intensive care unit (ICU) for monitoring/care.

### 2.4. Statistical Analyses

All statistical analyses were conducted using IBM SPSS© (version 27, IBM Corp., Armonk, NY, USA) or GraphPad Prism (version 9.2.0, San Diego, CA, USA). Mean values +/− standard deviation (SD) were calculated, and a Student’s *t*-test was performed for ICP values; Bonferroni corrections were applied as appropriate. A *p*-value of ≤0.05 was defined as statistically significant.

## 3. Results

**Figure 3:**
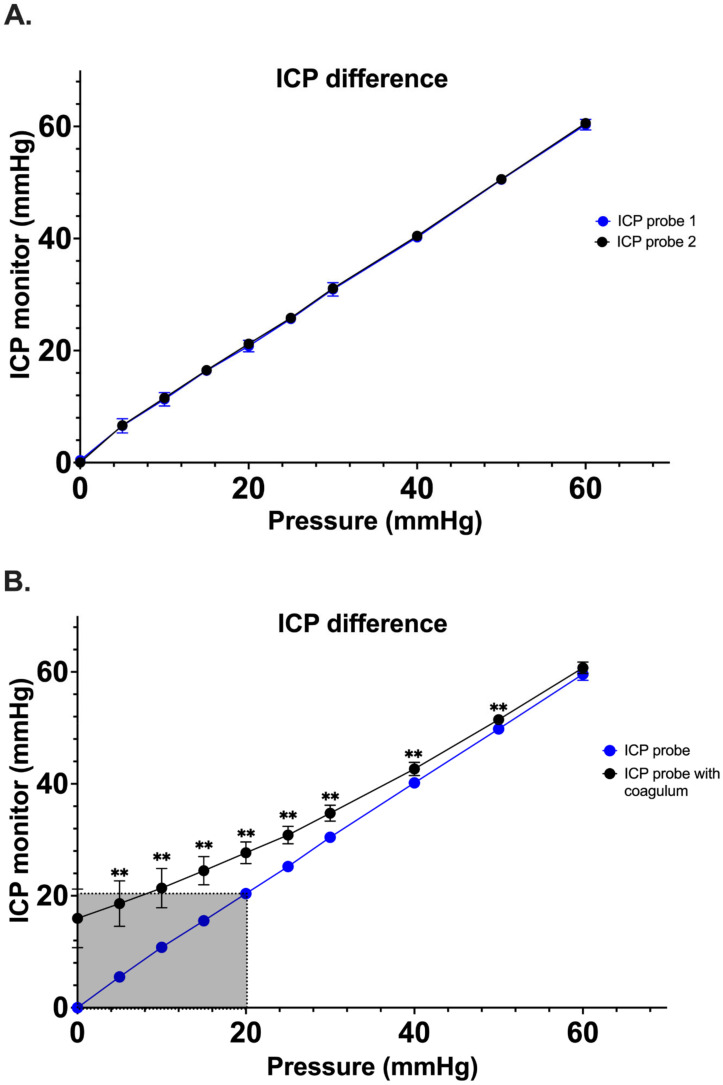
(**A**). The correlation of pressure measurements with the increase in the pressure within the drain system. No significant differences between the probes were observed. (**B**). Significant differences between control and clot-coated probes were observed between baseline (0 mmHg) and 50 mmHg (** *p* < 0.001). In shaded part, the significant difference can be observed up to the critical threshold value of 20 mmHg. At 60 mmHg, there was no significant difference between those measurements. ICP, intracranial pressure.

**Figure 4:**
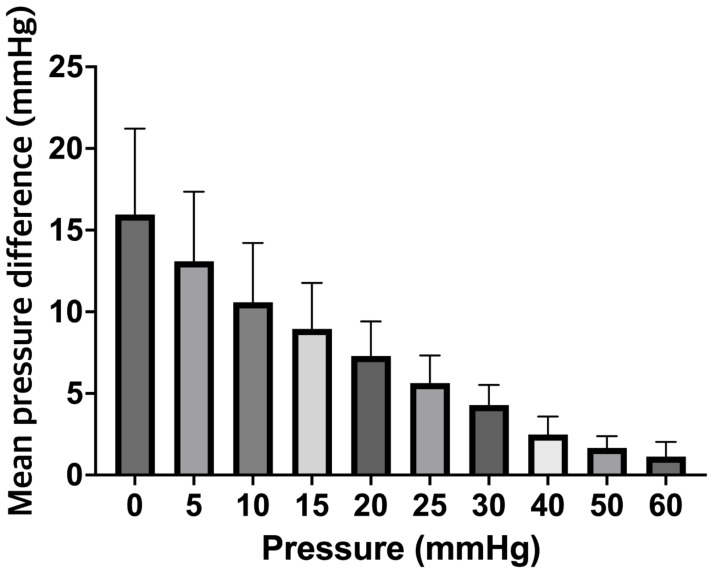
Bar chart depicting the mean ICP differences between control and clot-coated ICP probes depending on the pressure in the drain system. ICP, intracranial pressure.

Simultaneous pressure measurements by two control Spiegelberg-3PN air-pouch ICP sensors demonstrated good correlation with no significant difference having been noted between the pressure values ascertained between 0 and 60 mmHg (n = 5; range 0–1.6 mmHg) (Figure 3A). Once the experimental system was optimized, a secondary study comparing ICP probes coated with clotted blood vs. control ICP probes was performed (n = 10). At 0 mmHg, the measured mean pressure of the ICP + clot probe was 16.0 ± 5.3 mmHg as compared to a mean pressure of 0 ± 0 mmHg of the control ICP probe (*p* < 0.001). Interestingly, a significant difference between both probes was observed up to 50 mmHg (51.5 ± 1.0 mmHg vs. 49.8 ± 0.4 mmHg, *p* < 0.001) (Figure 3B). It is also important to note that we observed falsely high mean ICP values over a range of clinically relevant ICPs for the ICP + clot probe. For example, the control probe correctly displayed a value of 10 mmHg when subjected to a pressure of 10 mmHg, while the ICP + clot probe displayed values of 21.4 ± 3.5 mmHg; these differences are illustrated in Figure 4.

**Figure 5:**
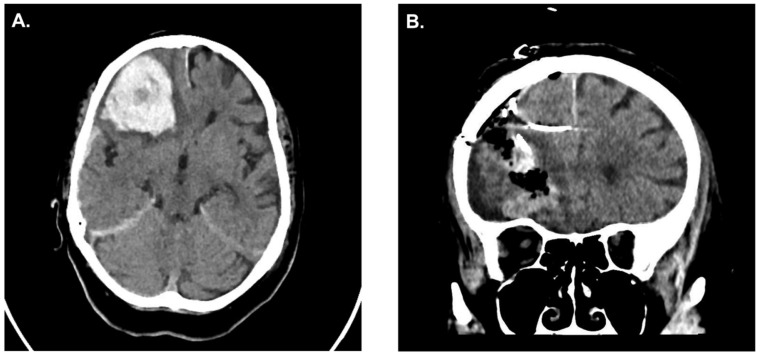
(**A**) Preoperative CT scan of a patient with right frontal intracerebral hemorrhage. (**B**) Postoperative CT scan showing hematoma evacuation with simultaneous ICP monitoring. ICP, intracranial pressure.

**Figure 6:**
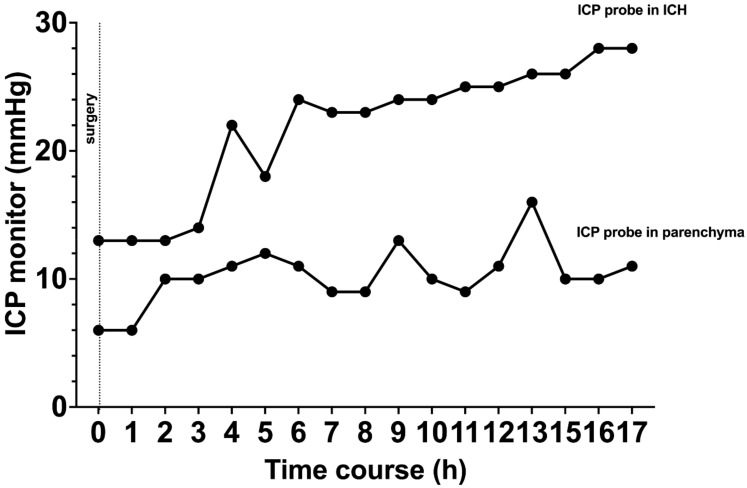
Simultaneous measurements of ICP probes located within the hematoma cavity and the parenchyma after hematoma evacuation. ICP, intracranial pressure; ICH, intracerebral hemorrhage.

In the clinical pilot, similar results were observed in our patient that underwent simultaneous ICP measurement; the patient presented with a right frontal ICH and the indication for surgical evacuation was made (Figure 5A). After hematoma evacuation, ICP probes were inserted (Figure 5B). The ICP probe located within the hematoma cavity consistently displayed higher ICPs values when compared to the ICP probe located in the adjacent parenchyma (Figure 6). No clinical or radiological signs of elevated ICP were observed during the course of observation in the neuro-ICU.

## 4. Discussion

The clinical relevance of ICP monitoring has been demonstrated in a myriad of studies and treatment parameters guided by ICP have been associated with improved outcome(s) and mortality [3,10,11]. A threshold of 22 mmHg has been defined as pathological by the Brain Trauma Foundation guidelines; such ICPs warrant an escalation of medical and/or surgical care [1]. Therefore, a simple and reliable measure of ICP is crucial as physicians/surgeons manage critically ill patients.

As noted above, certain classes of ICP probes require zeroing procedures prior to implantation. Further, such probes may drift (e.g., from −13 to 22 mmHg with the Camino device (Camino Laboratories, San Diego, CA, USA)) or an average difference of 10 mmHg of all recordings in Codman microsensors (Codman/Johnson & Johnson, Raynham, MA, USA) [8,12,13,14]. Given these concerns, the Spiegelberg air-pouch balloon-based ICP system has a number of pertinent advantages (i.e., an automated zeroing process after connection to the ICP monitor and the repeated hourly zeroing process correcting potential zero drift). In line with such advantages, the reliability of this class of ICP probe has been reported in several experimental and clinical studies [8,9,15]. Despite the noted benefits of the Spiegelberg ICP system and its correlation with gold standard ventricle measurements, herein we identified/highlighted a potential pitfall in the correct application/measurement related to probe placement. The air-pouch balloon-assisted sensor used in this study has a defined volume in the pouch, and 0.1 mL of air is pumped into the pouch during the zeroing process. Our clinical experiences and experimental studies suggest that clot adherence to the air pouch may lead to aberrant measurements. While the exact mechanism for such findings remains to be fully elucidated, we would posit that they related directly to perturbations in total pouch volume.

Given our initial clinical case and experimental findings, we felt it was extremely important to obtain prospective clinical data. The differences between ICP values for the probe located in the hematoma vs. adjacent parenchyma was even more extreme than expected. Given these data and the risks associated with inaccurate ICP measurements in neurocritical care, we feel it is imperative to make the following recommendations:

First, this class of ICP probe should not be placed in the regions prone to blood accumulation (e.g., a hematoma cavity or within epidural/subdural spaces). Second, the location of an air-pouch ICP probe should not be placed in the immediate proximity of other tissue that may affect the extension of the air-pouch (i.e., bone, dura mater, and/or the falx).

## 5. Limitations

We acknowledge that the experimental set-up employed is truly reductionist in nature and does not replicate the true complexity of an in vivo environment. In reality, the ICP probe would be surrounded by tissue and would experience continuous fluid exchange at physiologic temperature. Although our clinical pilot (N = 1 patient) yielded results that supported our experimental model, it is clear that future prospective studies will be required to validate and expand on this body of work.

## 6. Conclusions

The air-pouch balloon-assisted ICP probe is both a reliable and simple tool for ICP measurement; however, special attention should be given when placing the ICP probe.

## Data Availability

The datasets used and/or analyzed in the current study are available upon request.
